# Studies for Improving a Rat Model of Alzheimer’s Disease: Icv Administration of Well-Characterized β-Amyloid 1-42 Oligomers Induce Dysfunction in Spatial Memory

**DOI:** 10.3390/molecules22112007

**Published:** 2017-11-18

**Authors:** Ágnes Kasza, Botond Penke, Zsuzsanna Frank, Zsolt Bozsó, Viktor Szegedi, Ákos Hunya, Klaudia Németh, Gábor Kozma, Lívia Fülöp

**Affiliations:** 1 Department of Medical Chemistry, University of Szeged, Dome square 8, Szeged H-6720, Hungary; kaszagi@gmail.com (Á.K.); frankzsu@gmail.com (Z.F.); bozso.zsolt@med.u-szeged.hu (Z.B.); szegv@yahoo.com (V.S.); klausz20@gmail.com (K.N.); fulop.livia@med.u-szeged.hu (L.F.); 2 LipidArt Research and Development Ltd., Temesvári krt. 62, Szeged H-6726, Hungary; akos.hunya@lipidart.com; 3 Department of Applied and Environmental Chemistry, University of Szeged, Rerrich Béla square 1, Szeged H-6720, Hungary; kozmag@chem.u-szeged.hu

**Keywords:** amyloid beta, AD rat model, icv administration, hippocampus, spatial memory, Morris water maze, long-term potentiation, Golgi staining

## Abstract

During the past 15 years, several genetically altered mouse models of human Alzheimer’s disease (AD) have been developed. These costly models have greatly facilitated the evaluation of novel therapeutic approaches. Injecting synthetic β-amyloid (Aβ) 1-42 species into different parts of the brain of non-transgenic rodents frequently provided unreliable results, owing to a lack of a genuine characterization of the administered Aβ aggregates. Previously, we have published a new rat AD-model in which protofibrillar-fibrillar Aβ1-42 was administered into rat entorhinal cortex (Sipos 2007). In order to develop a more reliable model, we have injected well-characterized toxic soluble Aβ1-42 species (oligomers, protofibrils and fibrils) intracerebroventricularly (icv) into rat brain. Studies of the distribution of fluorescent-labeled Aβ1-42 in the brain showed that soluble Aβ-species diffused into all parts of the rat brain. After seven days, the Aβ-treated animals showed a significant decrease of spatial memory in Morris water maze test and impairment of synaptic plasticity (LTP) measured in acute hippocampal slices. The results of histological studies (decreased number of viable neurons, increased tau levels and decreased number of dendritic spines) also supported that icv administration of well-characterized toxic soluble Aβ species into rat brain provides a reliable rat AD-model.

## 1. Introduction

Alzheimer’s disease (AD) is the most common form of dementia. AD is a neurodegenerative disease that begins with synaptic dysfunction, which results in the loss of dendritic spines and post-synaptic density, finally leading to the failure of neuronal networks [1]. The initial abnormal neuronal activity progressively triggers neuronal cell death [2] as a consequence of the disruption of many intracellular processes (e.g., protein folding and degradation, mitochondrial function, etc.) [3]. β-amyloid (Aβ) may play a crucial role in the initiation of AD.

AD demonstrates phenotypic (clinical, imaging and pathological) heterogeneity [4]. Plenty of hypotheses try to explain the etiopathology of the disease. Based on the time of onset, AD is classified into two types [5]. Early-onset AD (EOAD) typically develops before the age of 65 years. The other form, late-onset AD (LOAD), develops in patients older than 65 years. The production and clearance of Aβ is regulated by a large group of genes. The genetic background of AD is widely reviewed [6,7,8,9]. For references, see also http://www.molgen.ua.ac.be/admutations/.

From the different AD theories, only the Aβ hypothesis has survived the conflicting results of AD research. The original hypothesis states that accumulation of Aβ in the brain is the primary event that drives AD pathogenesis [10,11]. The Aβ protein (a heterogeneous mixture of peptides of 39–43 AA) is derived from the proteolytic cleavage of amyloid precursor protein (APP) by β- and y-secretases. Aβ accumulates in the brain mainly as extracellular plaques, but accumulation of intracellular Aβ also occurs in the early stage of AD [12,13].

According to Walsh, soluble oligomers and protofibrils initiate AD pathomechanism [14]. Other results emphasize the important role of Aβ oligomers, protofibrils and fibrils in Aβ toxicity [15]. Recently, the formation of soluble toxic oligomers is considered to be a key event in AD pathogenesis. Increasing evidence indicates that the low-molecular weight oligomeric pre-fibrillar aggregates are the most highly cytotoxic species [16]. The precise molecular mechanisms of AD are still not fully understood despite 30 years of very intensive research. It is clear that LOAD is a multifactoral disease with a complex genetic background.

The familial type EOAD has simple genetics: one of the three main AD proteins (the amyloid precursor protein (APP), presenilin-1 and -2 (PSEN1 and PSEN2)) have mutations. Protein dyshomeostasis and amyloid formation are central events of both forms of AD, neuroinflammation and vascular dysfunction may play crucial roles in the onset of LOAD.

There is no “natural” animal model of the disease due to a poor understanding of AD and the complexity of the human brain [17]. Pharmacological and genetic AD- models and different animal species (primates, dogs, rodents, etc.) have been used in AD experiments during the 25 last years [18,19]. Genetically modified (transgenic, knock-in and knock-out) animal AD models provide a powerful approach to understand AD pathogenesis and study the disease [20]. These models allow investigation of the early stages of the disorder. There are several *Caenorhabditis elegans* [21,22] and *Drosophila melanogaster* [23,24] models of AD [20], which are widely used in screening experiments. A great variety of first- and second-generation transgenic mouse models of AD have been developed during the last 20 years for studying the pathophysiological processes of the disease, as reviewed in [25,26,27,28,29,30,31]. The transgenic mouse and knockout models analyze certain aspects of AD pathology, allowing exploration of unknown territories and revealing new pathogenic possibilities [32]. Summaries of the most prominent mouse models of AD have been published very recently [31,33,34]. Transgenic rat models were also introduced [35,36], possessing the key histopathological features of human AD (amyloid plaques, neurofibrillary tangles) without widespread cell loss. However, they have had limited success in translation of these findings into the clinics. The most obvious divergence of transgenic animal models from human AD is the artificial nature of transgenic technology. Rodents do not develop AD. The normal in vivo concentration of Aβ might be in picomolar range, contrary to that in human AD brains that has nanomolar Aβ levels. In addition, rodent Aβ differs from human Aβ by 3 AA substitutions (R5G, Y10F and H13R) and these changes also might prevent amyloid aggregation. As a consequence, the introduction of at least one of the main human AD genes (APP, PS1, PS2 and ApoE) is mandatory to model the pathology in rodents [33]. The ideal transgenic model should mimic multiple aspects of the disease including its etiology and a time-dependent progression of the pathology, which involves similar structures and cells, alike in human pathology.

Other less expensive, pharmacological animal models have also been introduced during the last 20 years. AD is considered to be a synaptic failure [37] and Aβ oligomers induce synaptic dysfunction [38,39], thus Aβ peptides have been used in model experiments. Synthetic Aβ-oligomers impaired long-term memory after icv injection into mice [40,41,42,43,44]. (Aβ peptides after icv injections reach brain cells by CSF (cerebrospinal fluid) influx via the perivascular and glymphatic pathways [45]). Rats offer numerous advantages over mice for developing AD models. Rats are closer to humans than mice [46]. Larger body and brain size facilitates in vivo electrophysiology, neurosurgical procedures and neuroimaging. The resurgence of interest in the rat as the animal model of AD led to the use of different types of rat models [19]. Aβ peptides were also administered intrahippocampally into rat brains [47,48,49,50]. Intracerebroventricular injection of Aβ1-42 to rats and mice were used to model AD [51,52]. Intranasal delivery or microinjecting Aβ oligomers into the entorhinal cortex was also applied in rats [53,54]. Two recent articles have compared the different pharmacological and genetic models of AD used in drug discovery [55,56].

The crucial problem of the application of Aβ in rat models is the heterogeneity of the peptide samples [57]. Aβ exists in vitro and in vivo as a continuum of different oligomeric states, none of which are particularly stable. All forms of Aβ-derived oligomers are potentially neurotoxic. During the fibril formation, several coexisting species are formed, giving rise to a highly heterogeneous mixture. The most difficult problem of the use of Aβ1-42 microinjections is the structural heterogeneity of oligomeric samples. This heterogeneity may cause severe problems in the evaluation of the results of Aβ-injection animal models and reproduction of in vivo experiments.

In our laboratory, we have tried to work out a novel, robust AD rat model, using icv administration of oligomeric Aβ1-42. The aim of the present study was to develop a reliable and cheap method by administering well-characterized Aβ peptides into the brain of wild-type rats. Atomic force microscopy was used for the characterization of Aβ oligomers. We have used histochemical methods to track the distribution of fluorescently labeled Aβ and characterize various neurodegenerative markers (number of viable neurons and dendritic spines, levels of tau). We also show that oligomeric Aβ-treated rats exhibit impaired synaptic plasticity (decreased LTP level) and cognitive disturbances (memory and learning behavior changes).

## 2. Results

### 2.1. Pilot Experiment with Icv Administration of AMCA-Labeled Aβ1-42 Oligomers

**Figure 1:**
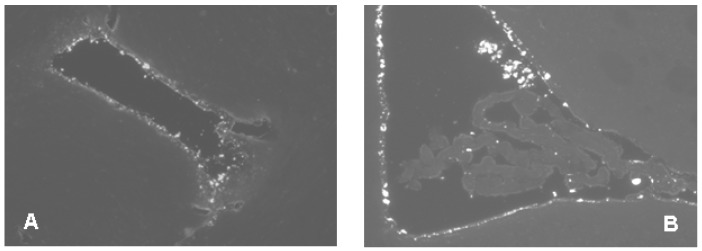
In this figure, icv administered fibrillar Aβ1-42 does not penetrate the ependyma and remains in the ventricles. Two representative examples of brain sections (**A**,**B**) after icv injected AMCA-labelled Aβ1-42 fibrils show the presence of the peptide in the ventricles at 1h after injection. Animals were injected bilaterally with 10–10 μL solution of AMCA fAβ1-42, and the surgical procedure was the same as described in Materials and Methods.

**Figure 2:**
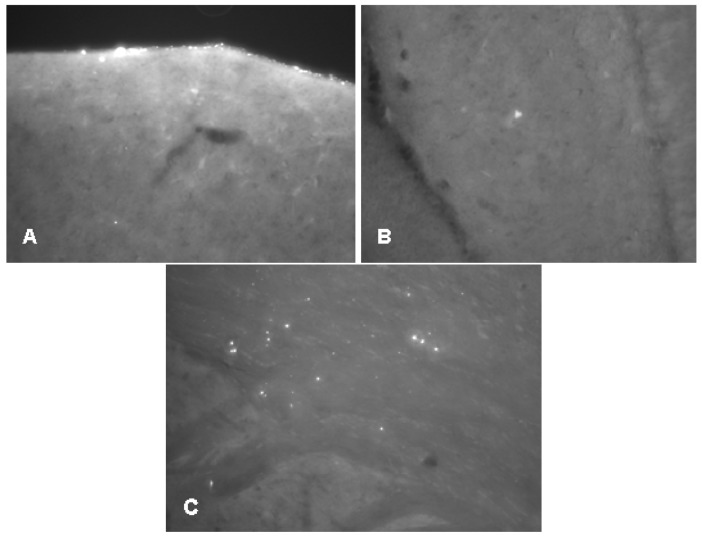
Oligomeric Aβ1-42 enters the brain parenchyma. Representative examples of brain sections after icv injected AMCA-labelled Aβ1-42 oligomers. (**A**): diffusion from the vehicle (5 min after injection) (**B**): signal in hippocampus (5 min after injection); (**C**): signals in brain parenchyma (60 min after injection). Animals were injected unilaterally with 7.5 μL solution, the surgical procedure was the same as described in Materials and Methods.

Before the icv administration experiments, we wanted to see whether Aβ1-42 oligomers could penetrate across the cerebroventricular wall. We have shown that the diffusion of the fibrilar form of Aβ1-42 is stopped by the ventricular wall, thus, an icv injection would be useless (Figure 1). Therefore our first aim was to demonstrate that oligomeric form of Aβ1-42 can reach the hippocampal (HC) region after icv injection. Using an unilateral injection of AMCA-labeled oligomeric Aβ1-42 (oAβ1-42) into the right lateral ventricle, we have found that the Aβ1-42 oligomers appear in the brain parenchyma: we could detect a few of fluorescent signs in the HC area as early as 5 min after injection (Figure 2A,B). The number of signs, namely of Aβ1-42 oligomers, was evidently higher around the HC area 60 min after the injection (Figure 2C), proving that the AMCA-labeled Aβ1-42 penetrates across the ependyma, or penetrates into the brain by the glymphatic flow [58].

### 2.2. Studies on the Neurotoxic Effect of Two Different, Icv Administered Aβ1-42 Aggregates into Rat Brains

We demonstrated that Aβ1-42 oligomers can reach the HC area after icv administration. The effect of different Aβ1-42 oligomers (24 h and 168 h aggregation time in 25 µM peptide concentration) was then studied. We analyzed the neuron viability with cresyl violet staining, tau level with tau-immunochemistry and the change of dendritic spine number was also measured. Our aim was to study if the Aβ1-42 oligomers show neurotoxic effects in the HC.

In the experiment, three groups of rats were used: the 24 h and 168 h aggregated Aβ1-42 treated groups in the same concentration (25 μM) as well as HCBS-treated control. Abbreviation used for oAβ-assemblies were: 24 h/25 μM and 168 h/25 μM.

#### 2.2.1. Histology

**Figure 3:**
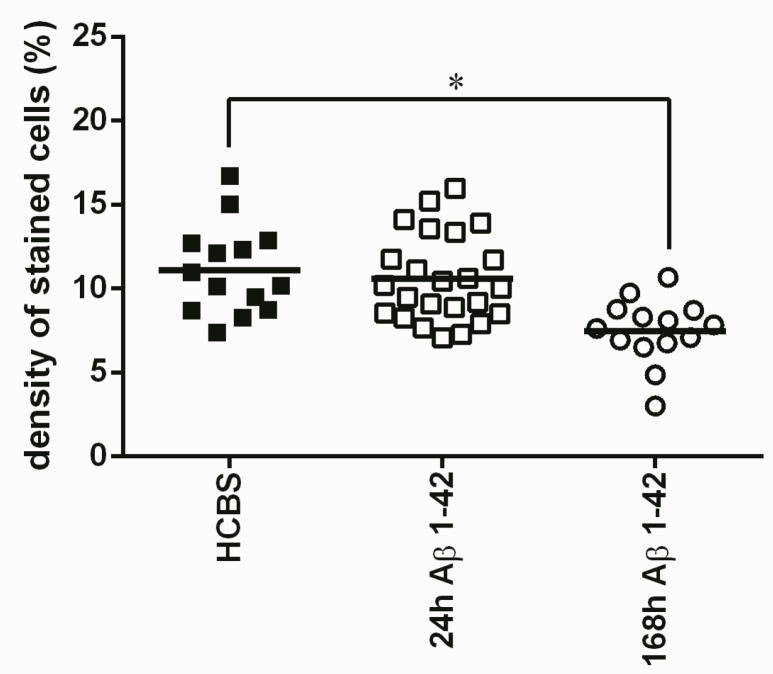
Cresyl violet staining of hippocampal slices after HCBS, 24 h aggregated and 168 h aggregated Aβ1-42 treatment (24 h/25 μM and 168 h/25 μM). Each dot represents the counted raw data, while horizontal bars indicate mean values. Significant difference in staining density were observed when compared HCBS vs. 168 h aggregated Aβ1-42 group (*p* = 0.001, *n* = 4, 2 slices/animal; *n* refers to the number of animals per group). Statistical significance was determined by one-way ANOVA, followed by Hochberg’s GT2 post hoc test. * Differences with a *p*-value < 0.05 were considered significant.

**Figure 4:**
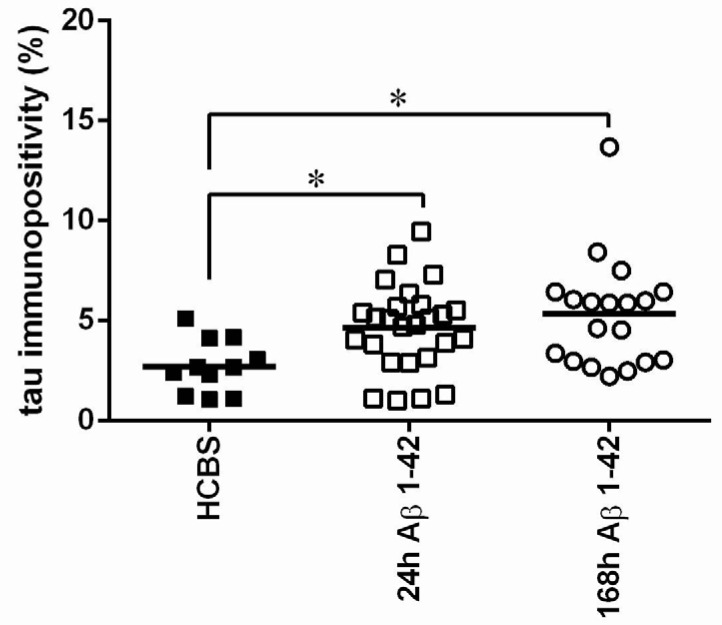
Tau-immunostaining of hippocampal slices after HCBS, 24 h aggregated and 168 h aggregated Aβ1-42 treatment (24 h/25 μM and 168 h/25 μM). Each dot represents the counted raw data, while horizontal bars indicate mean values. Significant difference in the number of tau-immunopositive cells were observed between HCBS vs. 24 h Aβ1-42 treated group and HCBS vs. 168 h Aβ1-42 treated group, (*p* = 0.042 and *p* = 0.007 respectively. *n* = 4, 4 slices/animal; *n* refers to the number of animals per group). Statistical significance was determined by one-way ANOVA, followed by Hochberg’s GT2 post hoc test. * Differences with a *p*-value < 0.05 were considered significant.

After icv administration of oAβ1-42 rats, significant differences exist between groups in the number of neurons. Significantly more viable neurons were counted in the HCBS treated group than in the 168 h/25 μM group (*p* = 0.001, *n* = 4 for each group, two slices/animal, Figure 3). (The 24 h/25 μM assembly did not cause a significant decrease.). Tau-immunochemistry showed neurotoxic effect in the Aβ1-42 treated groups, significantly more abnormally accumulated TNFs could be detected in both Aβ1-42 treated groups (24 h/25 μM and 168 h/25 μM) compared with the vehicle (HCBS) group (HCBS vs. 24 h/25 μM *p* = 0.042; HCBS vs. 168 h/25 μM oAβ1-42 *p* = 0.007, *n* = 4 for each group, four slices/animal, Figure 4).

**Figure 5:**
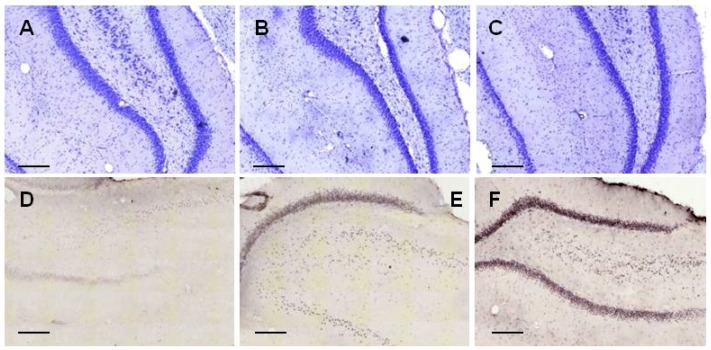
Representative coronal examples of hippocampcal sections stained with cresyl-violet (first row) to show the presence of neuron number (first row) and with tau-antibody to show the presence of abnormally aggregated NFTs (second row). (**A**,**D**): control group; (**B**,**E**): 24 h aggregated 25 μM amyloid treated group; (**C**,**F**): 168 h aggregated 25 μM amyloid treated group. Scale bar: 200 µm.

Representative examples of coronal HC sections (Figure 5) show the staining of neurons after 25 μM Aβ1-42 administration (A: control group, B: 24 h aggregation, C: 168 h aggregation assembly), and the abnormal aggregated NFTs (D: control group, E: 24 h aggregation, F: 168 h aggregation sample).

#### 2.2.2. Studying the Change of Dendritic Spine Density Using Golgi-Cox Impregnation

**Figure 6:**
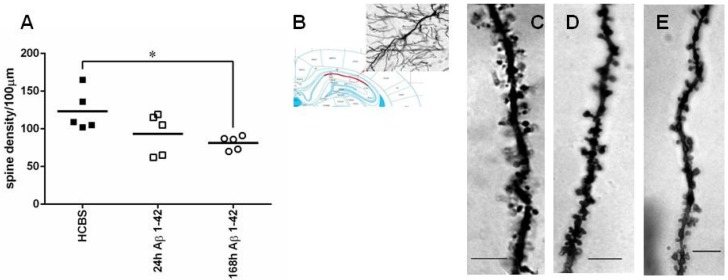
Representative examples of hippocampcal sections stained with Golgi-Cox method to show the presence of the prevailing changes in spine density after treatment with 24 h or 168 h aggregated Aβ1-42 (24 h/25 μM and 168 h/25 μM). (**A**): apical dendritic spine density. Each dot represents the counted raw data, while horizontal bars indicate mean values. Significant difference in spine densities was observed between HCBS and 168 h aggregated oAβ1-42 treated groups (*p* = 0.048, *n* = 2, 2–2 slices per group and 3–3 neurons per slice; *n* refers to the number of animals per group). Statistical significance was determined by one-way ANOVA, followed by a Games Howell post hoc test. * Differences with a *p*-value < 0.05 were considered significant; (**B**): 20× magnification of a CA1 subfield pyramidal neuron; (**C**): control (HCBS) group; (**D**): 24 h aggregated 25 μM Aβ1-42 treated group; (**E**): 168 h aggregated 25 μM Aβ1-42 treated group. Scale bar: 10 µm.

The same three groups of animals (24 h/25 μM; 168 h/25 μM oAβ1-42 and HCBS-treated control) were used as in the former experiments. We found significant differences in spine density comparing the 168 h aggregated Aβ1-42 treated to the control group. The 168 h/25 μM oAβ1-42 injected group had significantly less dendritic spines than the HCBS-treated control group (*p* = 0.048, Figure 6). There was no significant difference in the 24 h aggregated Aβ1-42 group compared with the controls. Representative photomicrographs demonstrated the difference between groups (Figure 6C–E, *n* = 2, 2–2 slices/group and 3–3 neurons per slice).

#### 2.2.3. Electrophysiological Studies

**Figure 7:**
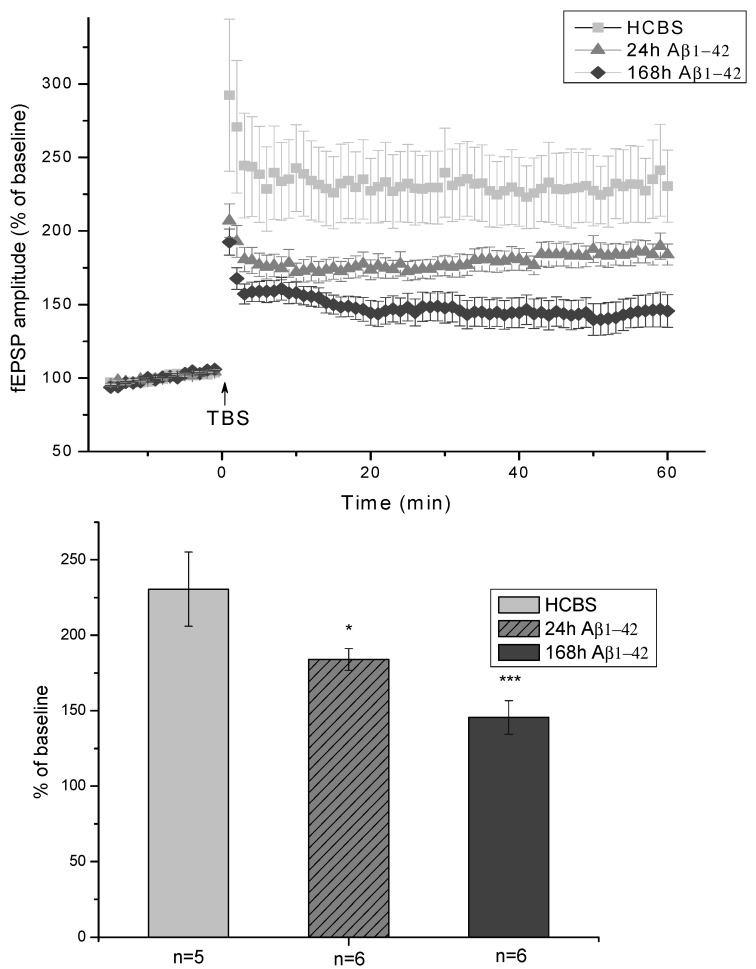
Shows the amplitude of fEPSPs normalized to pre-LTP control. The fEPSPs were recorded from the proximal stratum radiatum of CA1. The LTP of the 168 h oAβ1-42 treated animals showed robust impairment compared to HCBS treated ones, while the decrease was smaller in the group of 24 h oAβ1-42 injected rats. The histogram shows the level of LTP between 55 and 60 min post-TBS for each group. Error bars represent mean ± SEM. * *p* ≤ 0.05 and *** *p* ≤ 0.001.

Ex vivo electrophysiological recordings with multi-electrode array (MEA) were performed in acute hippocampal slices in artificial cerebrospinal fluid (ACSF). After establishing a stable baseline, LTP was elicited by applying a theta-burst stimulation (TBS) protocol and followed for an hour. The average of the peak-to-peak amplitudes of fEPSPs before the LTP induction was taken as 100% (Figure 7). The slices obtained from HCBS-injected animals showed robust potentiation after TBS (230 ± 24%; *n* = 5 slices). The two groups of icv injected animals treated with 24 h/25 μM and 168 h/25 μM oAβ1-42 aggregates showed impairment of LTP. The 24 h Aβ1-42 assemblies caused only a minor impairment (184 ± 7%; *n* = 6 slices), while the 168 h amyloid aggregates sled to a major disruption of potentiation (145 ± 11%; *n* = 6 slices, Figure 7).

### 2.3. Systematic Studies for Finding the Most Toxic form of the Aβ1-42 Oligomers

The influence of both the peptide concentration and the aggregation time on the toxicity of oAβ1-42 assemblies were studied in these experiments. Altogether, the effect of six different Aβ1-42 assemblies were studied in the biological experiments (see Table 1).

#### 2.3.1. AFM Studies of the Effect of the Concentration and the Aggregation Time on the Size of Aβ1-42 Oligomers

**Figure 8:**
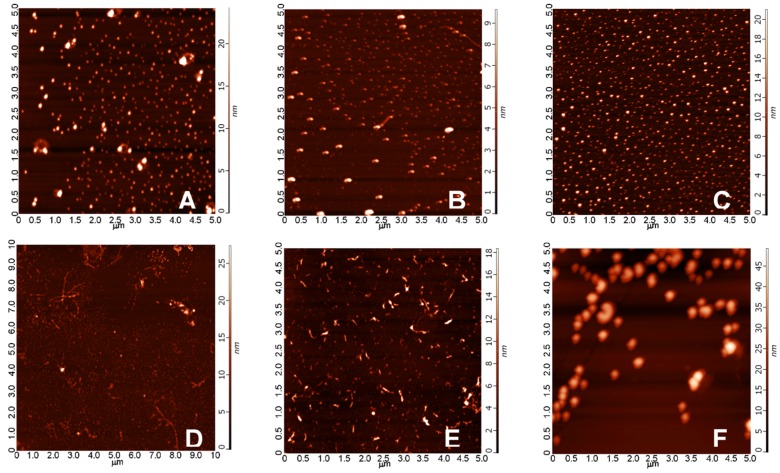
Morphology of Aβ1-42 oligomers observed on a mica surface in AFM experiments. Average heights of the aggregates after 24 h of aggregation were as follows: (**A**) 6.5 nm in a 25 μM solution; (**B**) 5.4 nm in 75 μM; (**C**) 10.3 nm in 200 μM. After 168 h, the following average heights were detected (**D**) 8.2 nm: in 25 μM; (**E**) 8.0 nm in 75 μM; (**F**) 21.5 nm in 200 μM. An elongated incubation resulted in the formation of protofibrillar aggregates besides the spherical ones, as it could be observed in images (**D**,**E**).

As the morphological characterization of a mixed oligomer preparation of Aβ1-42 is crucial for the better understanding of the biological effects exerted by the different types of oligomers, we conducted in vitro aggregation studies, in which different concentrations of Aβ1-42 were incubated in physiologic buffers for an elongated period of time. Morphology and size of the aggregates were studied by atomic force microscopy (AFM) in tapping mode. The representative images showed that, under the applied conditions, mainly spherical oligomers were formed after 24 h, the size of which did not depend considerably from the peptide concentration (Figure 8A–C) as average heights of the aggregates after 24 h of aggregation were as follows: (A) 6.5 nm in a 25 μM solution; (B) 6.5 nm in 75 μM; (C) 10.3 nm in 200 μM. After 168 h, besides the spherical oligomers, protofibrillar aggregates appeared at smaller concentrations (25 μM and 75 μM), while, in the extremely high 200 μM concentration, we could experience the massive formation of large round aggregates, presumably due to the strong steric hindrance between the monomers in the overcrowded aggregation environment. The detected average heights were (D) 8.2 nm in 25 μM, (E) 8.0 nm in 75 μM, and (F) 21.5 nm in 200 μM, respectively. Biological effectiveness of these different aggregates was further studied in consecutive biological experiments.

#### 2.3.2. Spatial Navigation in Morris Water Maze

**Figure 9:**
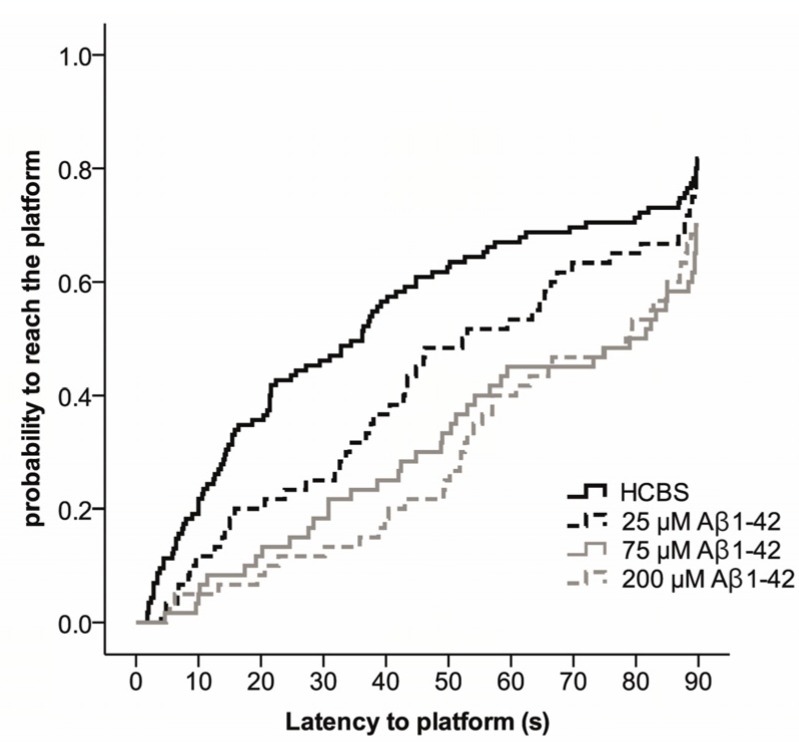
Effect of different aggregation concentrations (25, 75 and 200 µM) of synthetic Aβ1-42 (24 h aggregation time) on Morris water maze performance. The fitted survival curves using the Cox Proportional Hazard model represents the probability that animals find the platform during a trial, capped at 90 s. We compared HCBS vs. 25 µM oAβ1-42 (*p* = 0.219), HCBS vs. 75 µM oAβ1-42 (*p* = 0.003), and HCBS vs. 200 µM oAβ1-42 (*p* = 0.001) treatment groups using log-rank tests (*n* = 12/group, except the HCBS group, where *n* = 23).

**Figure 10:**
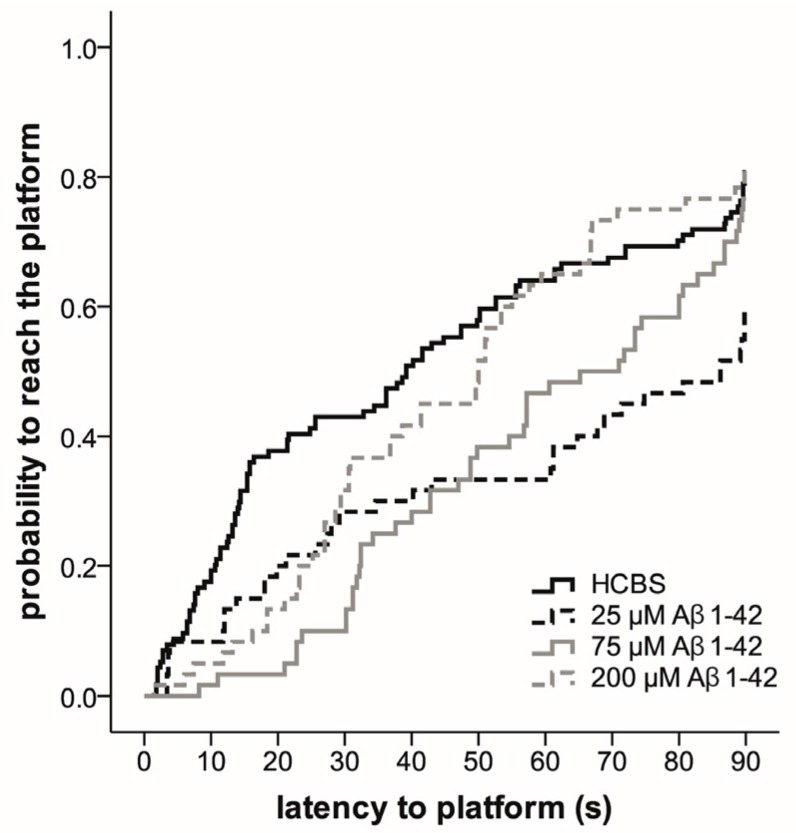
Effect of different aggregation concentrations (25, 75 and 200 µM) of synthetic Aβ1-42 (168 h time) on Morris water maze performance. The fitted survival curves using the Cox Proportional Hazard model represents the probability that animals find the platform during a trial, capped at 90 s. We compared HCBS versus 25 µM oAβ1-42 (*p* = 0.001), HCBS vs. 75 µM oAβ1-42 (*p* = 0.053), and HCBS versus 200 µM oAβ1-42 (*p* = 0.534) treatment groups using log-rank tests (*n* = 12/group, except HCBS group, where *n* = 23).

The Morris water maze (MWM) task was used to assess spatial learning and memory. MWM is one of the most commonly used experimental models for rodents to measure spatial learning and memory [59,60,61,62,63,64]. The total time spent in arena from first trials (time spent with searching the platform) was the most informative data. The results are represented in Figure 9 and Figure 10.

Compared to day one of testing, in the lower aggregation grade (24 h aggregation), the 75 µM Aβ1-42 treated *Group B* (*p* = 0.003) and 200 µM Aβ1-42 treated *Group C* (*p* = 0.001) rats were more likely to find the platform on day four (*p* < 0.001) and five (*p* < 0.001) than the HCBS treated group; however, the change was not significant on day two (*p* = 0.959) and three (*p* = 0.06). The probability to reach the platform was determined using the Cox Proportional Hazard model (Figure 9).

Compared to day one of testing, the rats in the 168 h/25 µM oAβ1-42 treated *Group D* (*p* = 0.001) found the platform more likely on day two (*p* = 0.002), three (*p* < 0.001), four (*p ≤* 0.001) and five (*p* < 0.001). The probability to reach the platform was determined using the Cox Proportional Hazard model (Figure 10).

#### 2.3.3. Histology

**Figure 11:**
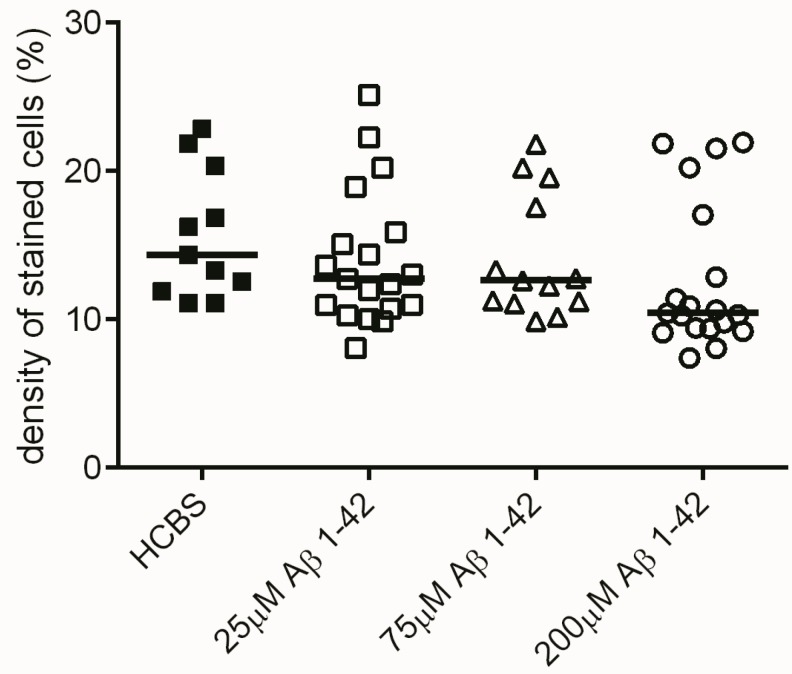
Cresyl violet staining (of hippocampal slices after treatment with different aggregation concentrations (25, 75 and 200 µM) of Aβ1-42 at a 24 h aggregation time. Each dot represents the counted raw data, while horizontal bars indicate median values. No significant difference in staining density, only a clear tendency of decrease was observed among the treatment groups compared to the HCBS group at the 0.05 significance level, *n* = 4, 2 slices/animal; *n* refers to the number of animals per group. Statistical significance was determined by the nonparametric independent-samples Kruskal-Wallis test.

**Figure 12:**
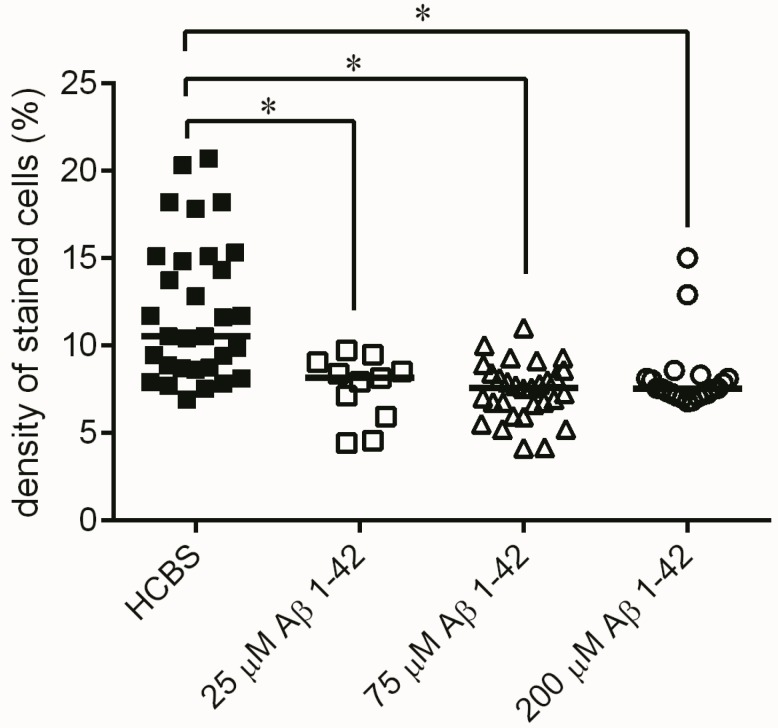
Cresyl violet staining of hippocampal slices after treatment with 168 h aggregated oAβ assemblies using increasing aggregation concentrations (25, 75 and 200 µM) of synthetic Aβ1-42. Each dot represents the counted raw data, while horizontal bars indicate median values. Significant differences in the staining density were observed between HCBS and the 25 µM Aβ1-42 treated group (*p* = 0.011), HCBS and the 75 µM Aβ1-42 treated group (*p* < 0.001), as well as between HCBS and the 200 µM Aβ1-42 treated group (*p* < 0.001), *n* = 4, 2 slices/animal; *n* refers to the number of animals per group. Statistical significance was determined by a nonparametric independent-samples Kruskal–Wallis test. * Differences with a *p*-value < 0.05 were considered significant.

Histochemical studies in the hippocampal region confirmed our behavioral results. Although no significant difference was found between the treatment groups in the number of viable neurons after administration of 24 h aggregated oAβ1-42 oligomers, there was a tendency that suggested that the increasing aggregation concentration of Aβ1-42 samples resulted in decreasing number of viable neurons in the examined area (*n* = 4; 2 slices/animal, Figure 11). Significant difference appeared between the 168 h aggregation time groups: significant loss of viable neurons was found in the hippocampal area between all oAβ1-42 treated groups (*D*–*F*) compared to the control HCBS group (HCBS vs. 25 μM *p* = 0.011; HCBS vs. 75 μM *p* < 0.001; HCBS vs. 200 μM *p* < 0.001; *n* = 4, 2 slices/animals, Figure 12).

**Figure 13:**
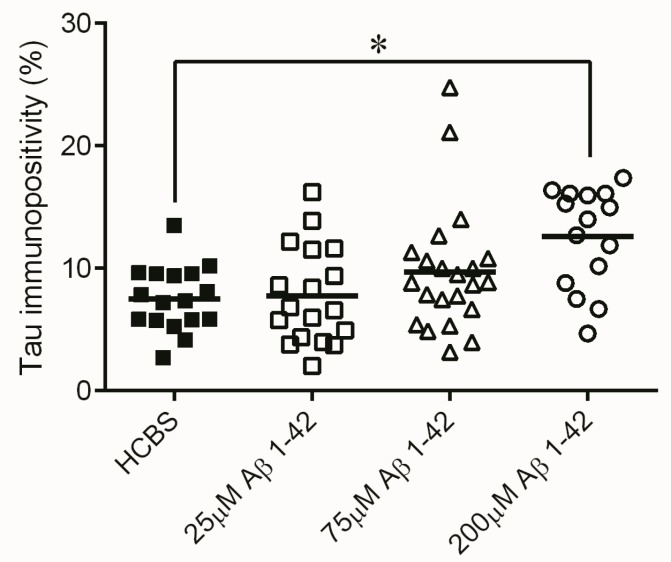
Tau-immunostaining of hippocampal slices after treatment with 24 h aggregated oAβ assemblies using increasing aggregation concentrations (25, 75 and 200 µM) of Aβ1-42 treatment. Each dot represents the counted raw data, while horizontal bars indicate mean values. Significant difference in the number of tau-immunopositive cells were observed only between the HCBS and the 200 µM Aβ1-42 treated group (*p* = 0.015, *n* = 4, 4 slices/animal; *n* refers to the number of animals per group) Statistical significance was determined by one-way ANOVA, followed by Hochberg’s GT2 post hoc test. * Differences with a *p*-value < 0.05 were considered significant.

**Figure 14:**
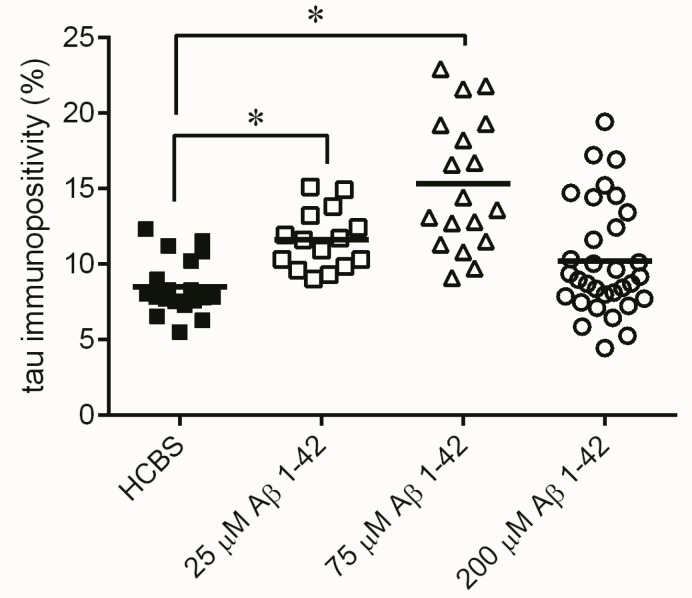
Tau-immunostaining of hippocampal slices after treatment of 168 h aggregated Aβ assemblies using increasing aggregation concentrations (25, 75 and 200 µM) of synthetic Aβ1-42. Each dot represents the counted raw data, while horizontal bars indicate mean values. Increasing number of tau immunopositive cells were observed comparing the HCBS and the 25 µM oAβ1-42 treated (*p* < 0.001), as well as the HCBS and the 75 µM oAβ1-42 treated groups (*p* < 0.001). There is no significant difference between HCBS and 200 µM oAβ1-42 treated groups (*p* = 0.284), *n* = 4, 4 slices/animal; *n* refers to the number of animals per group. Statistical significance was determined by one-way ANOVA, followed by a Games–Howell post hoc test. * Differences with a *p*-value < 0.05 were considered significant.

Monitoring the presence of abnormally accumulated TNFs and comparing to the control group, significantly higher number of tau-immunopositive cells were observed in the 24 h/200 µM treated *Group C* (*p* = 0.015, *n* = 4, 4 slices/animal, Figure 13), the 168 h/25 µM treated *Group D* (*p* < 0.001, Figure 14) and the 168 h/75 µM *Group E*, (*p* < 0.001, *n* = 4, 4 slices/animal, Figure 14) treated groups.

**Figure 15:**
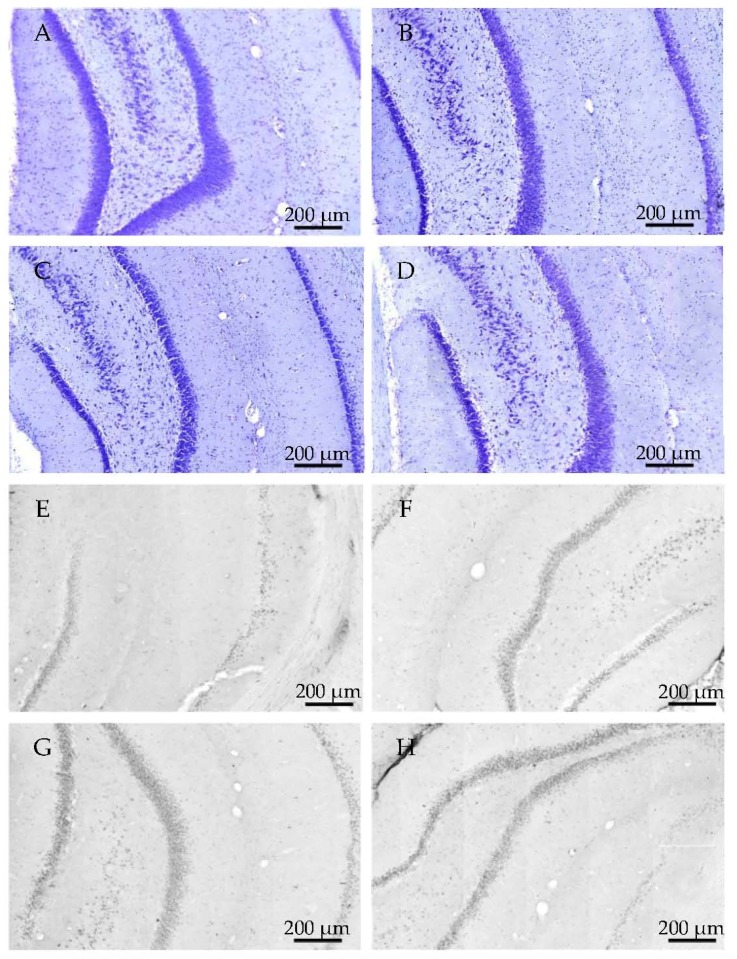
Representative examples of hippocampcal sections in 24 h aggregation groups, using cresyl-violet staining, show the change of viable neuron number (**A**–**D**). Immunochemistry with tau-antibody shows the presence of abnormally aggregated NFTs (**E**–**H**). (**A**,**E**): control group; (**B**,**F**): 25 μM concentration; (**C**–**G**): 75 μM concentration; (**D**–**H**): 200 μM concentration.

Representative examples of coronal HC sections show neurons after 25, 75 and 200 μM oAβ1-42 administration with 24 h aggregation time (Figure 15A–D) using cresyl violet staining. The abnormal aggregated NFTs are demonstrated in Figure 15E–H).

**Figure 16:**
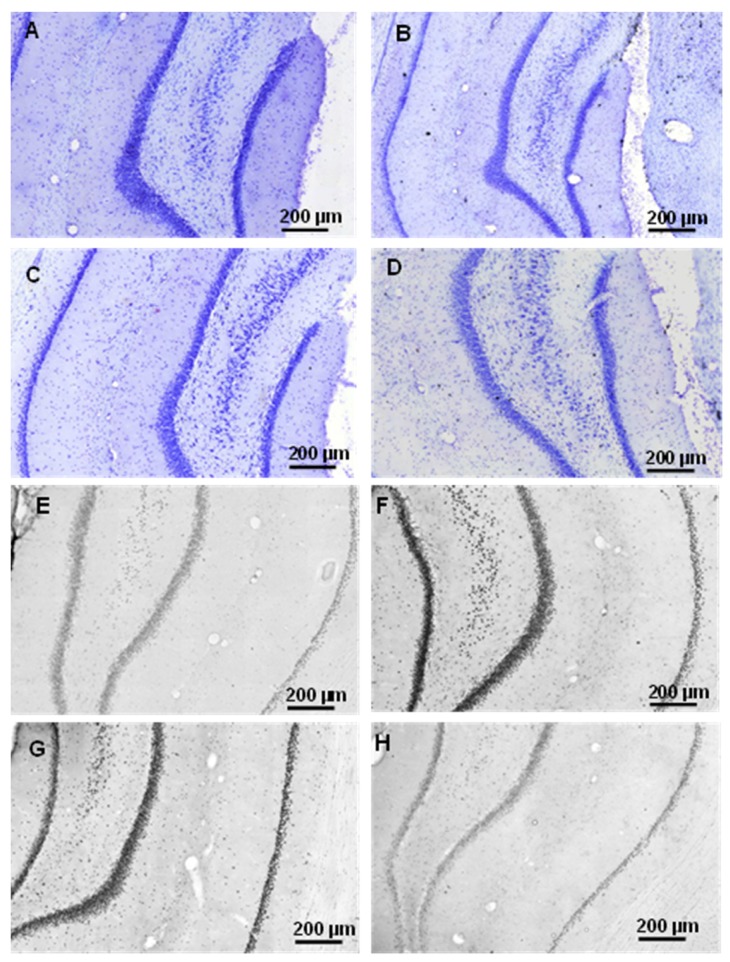
Representative examples of hippocampcal sections in 168 h aggregation time groups using cresyl-violet staining to show the decrease of viable neuron number (**A**–**D**). Immunochemistry with tau-antibodies shows the increase of abnormally aggregated NFTs (**E**–**H**). (**A**,**E**): control group; (**B**,**F**): 25 μM concentration; (**C**–**G**): 75 μM concentration; (**D**–**H**): 200 μM concentration.

Representative examples of coronal HC sections show the presence of neurons after 25, 75 and 200 μM oAβ1-42 administration with 168 h aggregation time (A–D), using cresyl violet staining. The abnormally aggregated NFTs are shown in Figure 16.

#### 2.3.4. Ex Vivo Electrophysiological Recordings with Multi-Electrode Array (MEA)

**Figure 17:**
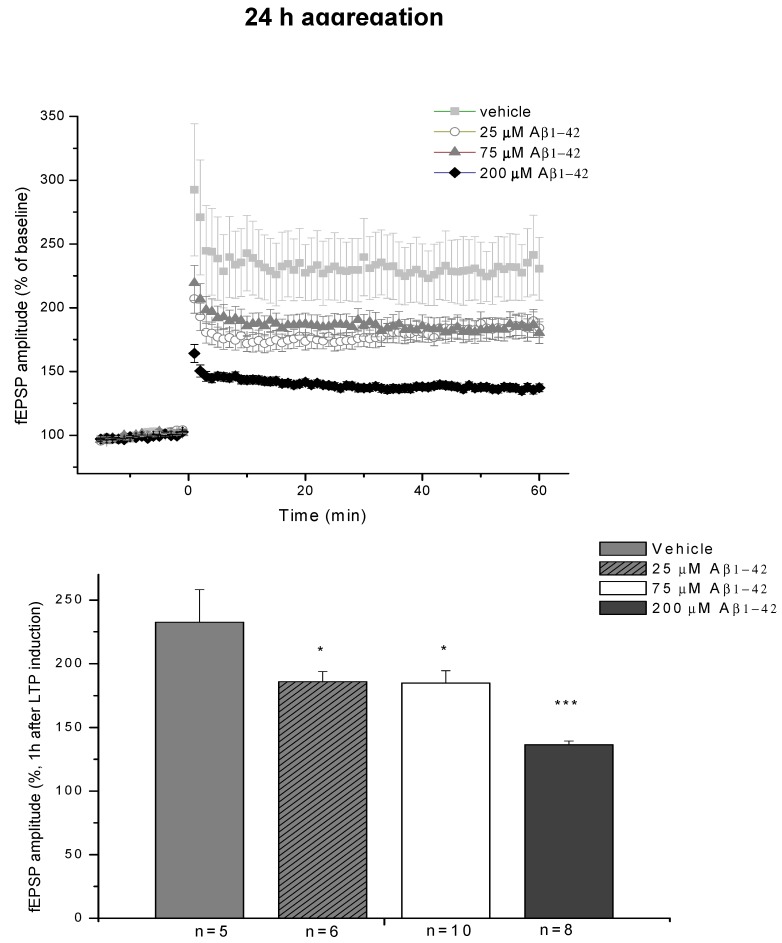
LTP impairment depends on the aggregation concentration. The effect of 24 h/75 μM, 24 h/25 μM and 24 h/200 μM oAβ samples on LTP in HC slices. The histogram shows the level of fEPSP potentiation between 55 and 60 min post-TBS. Error bars represent mean ± SEM. * *p* ≤ 0.05 and *** *p* ≤ 0.001.

The electrophysiological studies in the hippocampal region confirm our behavioral and immunohistochemical results. Slices were prepared from rats that had received icv administration of 24 h/25 μM (*Group A*) and 24 h/75 μM oAβ1-42 (*Group B*). Both oAβ1-42 samples caused reduced potentiation after LTP induction (168 ± 8% and 185 ± 10%, *n* = 6 and 10, respectively) compared to the HCBS treated group (233 ± 26%, *n* = 5, Figure 17). The 24 h/200 μM oAβ peptide (*Group C*) caused the greatest reduction of LTP level (136 ± 3%, *n* = 8, Figure 17).

**Figure 18:**
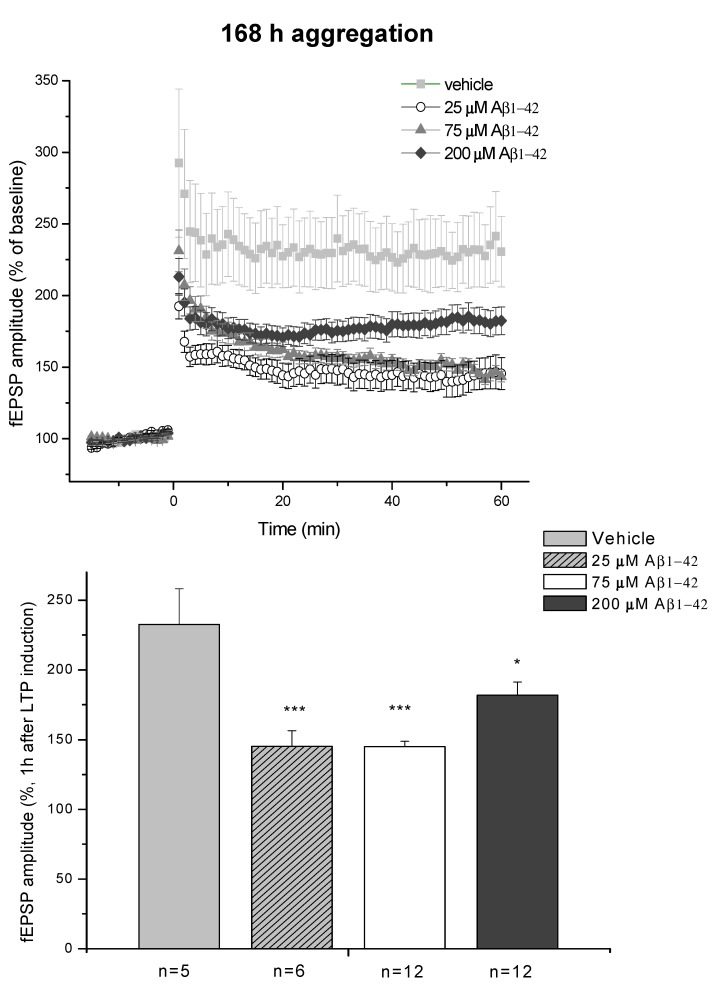
LTP impairment depends on the aggregation concentration of Aβ1-42. The effect of 168/25 μM, 168 h/75 μM and 168/200 μM oAβ1-42 samples in acute HC slices. The histogram shows the level of fEPSP potentiation between 55 and 60 min post-TBS. Error bars represent ± SEM. * *p* ≤ 0.05 and *** *p* ≤ 0.001.

The effect of the 168 h aggregates also showed concentration dependence (Figure 18). Similarly to the 24 h aggregation experiments, the effects of 25 μM (*Group D*) and 75 μM (*Group E*) peptide assemblies did not differ from each other, as they similarly reduced LTP level (145 ± 11%; *n* = 6 and 145 ± 4%; *n* = 12, respectively). In contrast, the 168 h/200 μM (*Group F*) oAβ aggregates caused much smaller reduction of LTP (182 ± 9%; *n* = 5 compared to HCBS group 232.5 ± 26%; *n* = 5).

## 3. Discussion

The main aim of the present work was a systematic study of the neurotoxic effects of icv administered oAβ assemblies in rat brain. Our principal aims were:

(1) Preparation of toxic Aβ1-42 oligomers from a precursor peptide (iso-Aβ1-42) and standardization of the method of the synthesis and characterization of oAβ1-42 assemblies.
(2) Measurement of the effect of different oAβ1-42 samples on neuron viability, NFT-formation, dendritic spine density, synaptic plasticity and spatial behavior in nontransgenic rats.
(3) Development of a novel rat model of AD using icv administration of well-characterized oAβ1-42 samples.

The role of amyloid plaques and oligomeric Aβ in AD etiopathology has been debated for a long time. Depositions of extracellular Aβ and the surrounding oAβ are considered as trigger signals to induce dendritic spine loss and synaptic dysfunction in AD. Aβ assemblies are synaptotoxic, and dendritic spine loss is strongly correlated with cognitive impairment in AD. Aβ has been shown to target synapses [65,66]. Experiments demonstrated that synapse dysfunction was triggered by Aβ oligomers [67]. Bilateral intrahippocampal (ihc) injections of fibrillar Aβ reduced neuronal density, increased the intensity of glial fibrillary acidic protein and caused behavior performance deficits [49,68]. Our former experiments also demonstrated that synthetic fAβ after ihc administration simultaneously decreased spatial learning ability in MWsM and reduced dendritic spine density in the rat hippocampus CA1 region [69]. As fAβ is a non-diffusible form and act only locally, the diffusible oligomeric Aβ assemblies (that surround fAβ) very probably affect the neurons and synapses in these experiments [70]. It is widely accepted that accumulation of soluble toxic Aβ at the synapse may be on the critical path to neurodegeneration [71]. Aβ-dependent disruption of neural cell adhesion molecules in AD hippocampus may contribute to synapse loss [72,73].

In our former studies, we found that the aggregation grade of the oligomeric Aβ1-42 samples plays a crucial role in the toxicity [53,54]. We demonstrated that the aggregation grade can be standardized using controlled in situ preparation of Aβ1-42 oligomers from the Aβ-precursor isopeptide (iso-Aβ1-42), and oAβ1-42 assemblies can be physicochemically characterized [74]. This method was used in the present work for preparation of different oAβ1-42 assemblies in two series of experiments. Different aggregation times (24 h and 168 h) and peptide concentrations (25 μM, 75 μM, 200 μM) were used, and the AFM method was applied for characterization of oAβ assemblies. 

In the first experiment, one peptide concentration (25 μM) and two different aggregation times (24 h and 168 h), in the second experiment three peptide concentrations (25 μM, 75 μM and 200 μM) and two different aggregation times (24 h and 168 h) were used. (The samples of the oAβ1-42 were signed as 24 h/25 μM, 168 h/25 μM, 24 h/75 μM, 168 h/75 μM, 24 h/200 μM, 168 h/200 μM.). The morphology of some of the oAβ1-42 assemblies is shown in Figure 8.

A pilot study (Figure 1 and Figure 2) demonstrated that AMCA-labeled fibrillary Aβ1-42 remained in the ventricles after injection, but AMCA-oAβ1-42 penetrated across the ependyma or entered the brain parenchyma by the glymphatic flow.

The results of the first experiments are shown in Figure 3, Figure 4, Figure 5, Figure 6 and Figure 7. There was a considerable difference between the effects of the two oAβ1-42 samples: the 168 h/25 μM sample showed significant change in neuronal viability (*p* = 0.001, Figure 3), increase in NFT-level (*p* = 0.007, Figure 4 and Figure 5), decrease of dendritic spine density (Figure 6, *p* = 0.048) and robust impairment of LTP (Figure 7, *p* < 0.001). The effects of 24 h/25 μM sample were not significant in the viability test (Figure 3 and Figure 5B), as well as in dendritic spine density measurement (Figure 6A). These results demonstrated that the aggregation time of Aβ1-42 plays a crucial role in the formation of toxic assemblies. In addition, 168 h aggregation time in 25 µM concentration resulted in the formation of toxic assemblies, while the 24 h samples gave less toxic aggregates. 

In the second series of experiments, the effect of six different oAβ1-42 samples were systematically studied. AFM studies of these oAβ1-42 samples demonstrated big differences in the size of the assemblies (Figure 8). The mean of the particle diameter was in the range of 6.5 and 21.5 nm. Besides the obvious differences in size, an altered morphology of the aggregates could also be observed, as protofibrils were formed together with the spherical oligomers in lower (25 and 75 µM) concentrations after 168 h. These oAβ1-42 assemblies were used in behavioral (learning and memory), histological and electrophysiological studies.

In this series of experiments, we examined whether these Aβ oligomers impair memory functions in rats, especially the spatial memory. We measured the effect of icv administered oAβ samples in MWM, a hippocampal learning and memory test, in which the animals have to learn the location of the hidden platform [60]. As HC appears to play a central role for establishment in long-term memory [75], we determined the mean of the first swimming session. The second trial every day was a potentiation for the animals to learn the exercise easier.

Finding of the hidden platform took a longer time for the Aβ-treated animals. After latency times of five days, Aβ1-42-treated animals exhibited significant differences compared to the control group. This finding is in accordance with the hypothesis that icv injection of iso-Aβ1-42 derived oligomers impair the spatial memory. The highest significance (greatest difference in latency times) compared with the control animals was exhibited by the *group C* (24 h/200 μM), where the particle size was 10 nm (Figure 8 and Figure 9), the *group D* (25 μM and 168 h), where the particle size exceeds 8 nm (Figure 8 and Figure 10).

Histological studies partly confirmed the results of behavioral experiments. Although the 24 h aggregates did not significantly decrease the number of viable neurons, there was a clear tendency for neuronal loss (Figure 11). The 168 h samples cause a significant decrease of viable neurons compared to the control (Figure 12).

Measurement of the abnormally accumulated NFTs in the HC slices gave interesting results (Figure 13): only the 24 h/200 µM oAβ1-42 assembly caused significant NFT accumulation. Aggregation time of 168 h in 25 and 75 µM concentrations resulted in the formation of toxic assemblies, but the 168 h/200 µM sample did not cause a change in the NFT level (Figure 14). These results were in good correlation with the size and mobility of oAβ assemblies (Figure 8). Representative examples showed the coronal HC sections, applied for neuron viability and NFT-accumulation measurements (Figure 15 and Figure 16).

Electrophysiological studies also supported the results of the behavioral experiments. Only the 24 h/200 µM oAβ1-42 sample caused a great reduction in LTP, although the samples with lower aggregation concentrations also showed a clear tendency for decreasing LTP (Figure 17). Finally, the concentration dependence in the 168 h groups was similar to the 24 h groups: the 25 and 75 µM oAβ1-42 samples caused robust and significant reduction, while the LTP reduction was much smaller in the 200 µM group (Figure 18). These results also correlate well with the size and viability of the oAβ assemblies.

Summarizing our studies, we found a simple correlation between the aggregation size and the mobility and toxicity of oAβ1-42. There was an optimal size of oAβ1-42 assemblies (between 8 to 10 nm height) that caused elevated toxicity (24 h/200 μM, 168 h/25 μM and 168 h/75 μM aggregates, (Figure 9, Figure 10, Figure 12, Figure 13, Figure 14, Figure 17 and Figure 18). Too small and too big aggregates (24 h/25 μM, 24 h/75 μM, 168 h/200 μM) were less toxic or nontoxic (Figure 9, Figure 11, Figure 13, Figure 14, Figure 17 and Figure 18). We assume that the size and different conformations, resulting in altered morphology, together are responsible for the enhanced toxicity. In addition, the peptide conformation within the oAβ1-42 samples is unknown, and we suppose that the toxic samples not only have similar particle size, but also structural similarity, containing oligomeric and protofibrillar Aβ1-42 species.

The connection between amyloid aggregation, cellular toxicity and the biochemistry of neurodegeneration has been a challenge, and the molecular details are more or less unknown. Determining the biophysical properties and conformational variety of a single species of amyloid peptides/proteins represents a high experimental challenge. The main problem is the heterogeneous nature and the nanoscale dimensions of the amyloid assemblies. Circular dichroism and infrared spectroscopy are bulk techniques and cannot characterize the inner properties of the aggregates at the single species level [57]. 

Taken together, the suitability of a new rat model was demonstrated. Our aim was to work out a novel, robust AD rat model using icv administration of well characterized oligomeric Aβ 1-42. Such kind of model has several advantages compared to the existing mice and rat models:

(1) Mice are typically more variable in their behavior than rats; thus, we were able to use fewer animals for getting significant results in rats than in mice in MWM and other behavior experiments.
(2) Rats are physiologically, genetically and morphologically closer to humans than mice [46]. Their larger body and brain size facilitate neurosurgical procedures, neuroimaging and in vivo electrophysiology.
(3) Our research group also successfully used intranasal delivery of human Aβ 1-42 for rat brain targeting, but intranasal administration is complicated and very time-consuming.
(4) The recent method is a short-term model: it is possible to get pathology and behavior changes within two weeks after the icv administration into rats.
(5) Several laboratories have used short human Aβ fragments (e.g., Aβ25–35) in rat experiments; however, these kinds of short Aβ peptides are not natural metabolites of Aβ degradation, only aggregation-prone synthetic products.
(6) Other laboratories (e.g., [49]) used a rat model of AD injecting fibrillary Aβ1-42 into the rat brain. In this model, the injected fibrillar Aβ aggregates form a deposit in the brain and a long time is necessary for destabilization and disintegration of the assemblies to diffusible toxic Aβ-oligomers. The use of icv administration of toxic oAβ1-42 samples is advantageous, and the optimal size of the Aβ1-42 assemblies is 8 to 10 nm height.

Our current study demonstrated that icv administration of oAβ assemblies or Aβ protofibrils of definite size and structure into rats decreased cell viability and dendritic spine density, increased NFT formation, disturbed synaptic plasticity and impaired the learning and spatial behavior of the animals. Our results improve the “icv-administered Aβ” rat model using well-characterized Aβ1-42 oligomers.

## 4. Materials and Methods

### 4.1. Preparation of Aβ1-42 Peptide Samples and Different Oligomeric Assemblies

#### 4.1.1. Preparation of Different Aβ1-42 Oligomeric Assemblies

The oligomeric Aβ1-42 peptide was synthesized in the following way: the Aβ peptide precursor iso-Aβ1-42 was synthesized by Fmoc-chemistry and transformed at neutral pH to Aβ1-42 by O→N acyl migration in a short period of time, resulting in a water soluble oligomeric mixture of Aβ1-42 oligomers as previously described [74]. Synthetic iso-Abeta peptide was pre-treated with HFIP in order to facilitate its oligomerization, and to standardize its aggregation. The aggregation grade of these oligomers thus formed was standardized. In these studies, the soluble Aβ1-42 oligomeric peptide samples were freshly prepared by incubating the oligomeric Aβ1-42 peptide on 37 °C in PBS at pH 7.4 for different time courses (24 h and 168 h) at the concentrations of 25, 75 and 200 μM, respectively. Samples were sonicated in normal bath sonicator for 5 min. Before use, each sample was subsequently diluted to the final concentration of 10 µM, and 15 µL (2 × 7.5 µL) of these solutions were injected into rat brain hemispheres (see Surgery). The amount of Aβ1-42 injected at the concentration of 10 µM in each case equaled 50 pmol or 225 ng of Aβ peptides.

The fluorescent labeled Aβ peptide (AMCA-Aβ) was synthesized also in our research group [76].

#### 4.1.2. Atomic Force Microscopy Studies (AFM) of Aβ1-42 Assemblies

For the experiment, 10 μL of peptide solution were pipetted onto freshly cleaved mica (Muscovite mica, V-1 quality, Electron Microscopy Sciences, Washington, DC, USA). After 2 min, the samples were washed twice with 10 μL of distilled water and then dried with nitrogen gas. The AFM images were obtained using tapping mode on a NT-MDT Solver Scanning Probe Microscope (NT-MDT Spectrum Intruments, Moscow, Russia) under ambient conditions. AFM tips type PPP-NVHAuD-10 manufactured by NANOSENSORS (Neuchâtel, Schwitzerland) were applied with a nominal radius of curvature of 2 nm and 15 μm length. The non-contact silicon cantilevers having typical force constant of 42 N/m and resonance frequency of 278.8 kHz. Further information of the tip: material n^+^-silicon, resistivity 0.01–0.02 Ω cm, thickness 4.0 ± 1 μm, length: 125 ± 10 μm, width 30 ± 7.5 μm.

#### 4.1.3. Fluorescent Microscopy

For fluorescent microscopy study, rats (*n* = 2) received fibrillar form, and, in the other experiment, rats (*n* = 4) received oligomeric form and of AMCA (7-Amino-4-methylcoumarin-3-acetic acid)-labeled Aβ1-42 as a single dose in dilution of AMCA-Aβ1-42:Aβ1-42 2:7 ratio (concentrations: AMCA-Aβ1-42 16.7 μM; Aβ1-42 = 58.3 μM in total of 75 μM final peptide concentration). fAβ was administered bilaterally (10–10 μL per site), and oAβ unilaterally into the right cerebroventriculum (7.5 μL pro animal). 60 min after fAβ1-42, and 5 or 60 min after oAβ1-42 administration the rats were transcardially perfused (100 mL PBS, pH = 7.4). The brains were removed and cut to 30 µm thick sagittal sections. Sections were placed on glass slides, air dried, and mounted in Gel Mount (Biomeda, San Diego, CA, USA). Fluorescent signal was examined in the sections by a Nikon Eclipse TE2000 fluorescent microscope (Nikon, Tokyo, Japan) and photographed by a Spot RT digital camera (Diagnostic Instruments, Sterling Heights, MI, USA).

### 4.2. Treatment Groups

#### 4.2.1. Studies on Two Different Aβ1-42 Oligomers (24 h and 168 h Aggregation Time, Concentration 25 µM)

Subjects were divided into three groups: the control group (*n* = 12) was injected with hydrocarbonate buffered saline (HCBS) solution, the other two groups were injected with 24 h aggregated Aβ1-42 (*n* = 12) and a 168 h aggregated Aβ1-42 (*n* = 12).

#### 4.2.2. Systematic Studies for Finding the Most Toxic Form among Six Different Aβ1-42 Oligomers

oAβ samples were prepared in combination of three different Aβ1-42 aggregation concentrations (25, 75 and 200 μM) with two different aggregation times (24 h and 168 h). The experiments were divided into two divisions: oAβ samples of 24 h and 168 h, respectively (altogether six groups):

A/ Aβ1-42, 24 h aggregation time, c = 25 µM; mean particle diameter 6.5 nm,
B/ Aβ1-42, 24 h aggregation time, c = 75 µM; mean particle diameter 6.5 nm,
C/ Aβ1-42, 24 h aggregation time, c = 200 µM; mean particle diameter 10.3 nm,
D/ Aβ1-42, 168 h aggregation time, c = 25 µM; mean particle diameter 8.2 nm,
E/ Aβ1-42, 168 h aggregation time, c = 75 µM; mean particle diameter 8.0 nm,
F/ Aβ1-42, 168 h aggregation time, c = 200 µM; mean particle diameter 21.5 nm.

The control groups (*n* = 11 in the 24 h and *n* = 12 in the 168 h experiments) in both studies were treated with HCBS. For the statistical analysis, the mean of data of the two control groups were evaluated (*n* = 23). Table 1 summarizes the characteristics of Aβ-treated groups (A–F) to simplify further orientation.

#### 4.2.3. Surgery and Icv Administration of oAβ1-42

Before surgery, rats were deeply anesthetized by e.g., injection of ketamine (10.0 mg/100 g) and xylasine (0.8 mg/100 g) mixture, and were placed in a stereotaxic apparatus. A midline incision of the scalp was made, and the skull was carefully cleared from the skin and the muscles. After that two holes were drilled above the target regions. Every solution was injected icv with Hamilton syringe bilaterally, into each hemisphere. Furthermore, a 7.5 μL solution was injected per site (1.5 μL/min). The coordinates were from bregma: AP: −1.0; ML: ±1.5; DV: −4,5. [77]. The animals were treated after the surgery with antibiotics and analgesic.

### 4.3. Spatial Navigation of Rats in a Morris Water Maze (MWM)

#### 4.3.1. Experimental Animals and Housing

Adult male Charles River-Harlan rats (Domaszék, Hungary) were the subjects of the experiments, weighing 250–300 g before surgery. After arrival, the animals were housed under constant temperature and lighting conditions (23 °C, 12:12 h light/dark cycle, lights on at 7:00). The rodents had free access to food and water throughout the experiment. After arrival, the animals were gently handled by daily measuring. Experiments were performed in accordance with the Hungarian Health Committee and the European Communities Council Directive of 24. November 1986 (86/609/EEC). Formal approvals to conduct the experiments have been obtained from the Animal Experimentation Committees of the University of Szeged and of the Biological Research Center, and from the local authorities (XVI/03835/001/2006).

#### 4.3.2. Morris-Water Maze Experiments

Animals were trained in open-field water maze (diameter: 180 cm) filled with water (23 ± 1 °C) that was made opaque with milk. The pool was divided into four virtual quadrants, and the invisible platform (diameter: 10 cm) was submerged in the middle of one of the four quadrants. Around the pool, there was a black curtain. The animals were allowed to swim for 5 days, twice a day and launched from four different starting points. They were placed into the water facing the wall of the pool and were given 90 s to find the platform and 15 s to stay on it. Animals that did not find the platform were gently guided and placed on it. The escape latency data were calculated automatically by a video tracking system (EthoVision 2002, Noldus Information Technology, Wageningen, The Netherlands). The means of the data (+SEM) from the first swimming sessions were used for statistics.

### 4.4. Histology

After the Morris water maze test, the animals were deeply anesthetized and transcardially perfused with 150 mL 4 °C phosphate-buffered saline solution (PBS), followed by 250 mL 4 °C paraformaldehyde solution (4% in phosphate buffer, pH 7.4). The brains were removed and postfixed for 24 h in the same fixative (4 °C), and subsequently cryoprotected in 30% sucrose solution for 72 h (4 °C). Brains were cut on a cryostat to 30 μm hippocampal coronal sections, and the slices were collected and stored at 4 °C in PBS for free floating histochemistry.

#### 4.4.1. Cresyl Violet (Nissl) Staining

The cresyl violet staining is used for neuronal tissue, the stain binds to the acidic components of the neuronal cytoplasm, showing the number of viable neurons. Slides were stained into the filtered 1% cresyl violet solution for 5 min and dehydrated subsequently in 50%, 70%, 95%, and twice in 100% ethanol for 1 min each. Slides were finally placed in xylene for another 10 min and coverslipped.

#### 4.4.2. Tau-Immunohistology

To visualize the presence of neurofibrillars tangles, we used human PHF-tau Mab (clone AT100) primer antibody at 1:800 dilution in PBS (pH 7.4) for immunostaining.

After quenching of endogenous peroxidase activity and a blocking step, the sections were incubated overnight at 4 °C with the primary antibody in the presence of 20% goat serum and Triton X-100 0.2%. On the following day, the sections were washed in PBS and incubated 1 h at room temperature with the second biotinylated goat anti-rat antibody (Vector Laboratories, Burlingame, CA, USA, 1:400). The next step was a 1-hour incubation with avidine-biotin complex (Vectastain Elit ABC Kit, Vector Laboratories, Burlingame, CA, USA; 1:400) and detection with nickel-enhanced 3,3′-diaminobenzidine. After immunostaining and washing, all sections were mounted on gelatin-coated slides, air-dried, dehydrated and coverslipped with DPX, a synthetic resin mounting media for histology (Fluka BioChemika, Buchs, Switzerland). Digital photographs were taken by a digital slide scanner (Mirax Midi, Carl Zeiss, Hungary); for the analysis, we used the Histoquant program (3DHistech, Budapest, Hungary).

### 4.5. Quantification of Dendritic Spine Density Using Golgi Impregnation

The FD Rapid GolgiStainTM Kit (FD NeuroTecnologies, Consulting & Services, Inc., San Diego, CA, USA) was used (*n* = 6, 2–2 slices per group and 3–3 neurons per slice) for measuring changes of dendritic spine density in the hippocampal CA1 area.

Experimental animals were deeply anesthetized before the brain was removed from the skull. The brains were removed as quickly as possible and handled carefully to avoid damage or pressing of the tissue. The tissue was immersed in the impregnation solution (A + B solution) and stored at room temperature for 2 weeks in the dark. The brains were transferred into another solution (C) and stored at 4 °C in the dark for at least 48 h. In addition, 100 µm coronal sections were cut with microtome (Zeiss Microm HM 650 V, Carl Zeiss AG, Oberkochen, Germany). Sections were mounted on gelatin coated glass slides. After the staining procedure and dehydration, the slides were covered with DPX (VWR international).

The Golgi sections were studied by inverse light microscope, using oil-immersion objectives. The spine density of the proximal apical dendrite area was analyzed (100–200 μm from soma). One segment (100 µm in length) from a second-third-order dendrite protruding from its parent apical dendrite was chosen in each examined neuron for spine density quantification, as described by [78]. The dendrites were selected under a 100× oil immersion lens and the images (600×) of these apical dendrites were captured through a CCD camera (1600 × 1200 pixel) connected to a light microscope (Olympus Vanox-T AH-2, Olympus Optical CO, LTD., Tokyo, Japan) and a computer. Serial images were made from each dendrite in the whole of the analyzed segment. The captured multiple photomicrographs from one dendrite were then stacked into one file. To stack the images, the Image- Pro-Plus image analysis software (IPP; Media Cybernetics, Silver Springs, MD, USA) was used. Measurement of the spine density was performed by two independent experimenters to blind the analysis.

### 4.6. Ex Vivo Electrophysiological Studies

#### 4.6.1. Stimulation Protocols

Using standard procedures, 350 µm thick transverse acute hippocampal slices were prepared from the brain using a McIlwain tissue chopper (Campden Instruments, Loughborough, UK). Slices were incubated in carbogenated standard artificial *cerebrospinal fluid* (ACSF; pH 7.4) at ambient temperature for at least 60 min that contained the followings in mM: NaCl, 130; KCl, 3.5; CaCl_2_, 2; MgCl_2_, 2; NaH_2_PO_4_, 0.96; NaHCO_3_, 24; d-glucose, 10. Individual slices were transferred to a 3D-MEA chip with 60 tip-shaped electrodes (40 µm in diameter and 50–70 μm in height, spaced by 200 µm, impedance at 1 kHz: 250–450 kΩ, noise level: 15–20 µV; purchased from Ayanda Biosystems, S.A., Lausanne, Switzerland). The surrounding solution was removed quickly and the slice was immobilized by a grid. The slice was continuously perfused with carbogenated standard ACSF (1.5 mL/min at 34 °C) during the whole recording session. Data were recorded by a standard, commercially available MEA (multi-electrode array) setup (Multi Channel Systems MCS GmbH, Reutlingen, Germany).

The Schaffer-collateral was stimulated by injecting a biphasic current waveform (± 100 µs) through one selected electrode at 0.033 Hz, while the rest of them could be used as recording electrodes. The positioning of the stimulating electrodes and that of the regions in the slices, compared to each other, were constantly synchronized during the various investigations. The peak-to-peak amplitudes of field excitatory postsynaptic potentials (fEPSPs) at the stratum radiatum of CA1 were analyzed. After a 30 min incubation period, the threshold and the maximum of stimulation intensity for evoke responses was determined. For evoking responses, 30% of the maximal stimulation intensity was used. When stable evoked fEPSPs were detected (for at least 20 min) LTP was induced, using a theta-burst stimulation (TBS) protocol applied at the maximum stimulation intensity. TBS comprised of 15 trains administered at 5 Hz, the individual trains contained 4 pulses separated by 10 ms. LTP was followed for an hour. 

For the statistical analysis of ex vivo recordings, the peak-to-peak amplitude of evoked fEPSPs recorded from the proximal part of stratum radiatum was calculated. The level of LTP was determined comparing the average of fEPSP amplitudes recorded in the last 5 min of the experiment to the baseline recording.

#### 4.6.2. Multi-Electrode Array (MEA) Recordings

Electrophysiological measurements followed the Morris water maze task, in which different icv injected Aβ1-42 was tested. TBS induced LTP recordings were performed using a multi-electrode array (MEA) setup.

After establishing a stable baseline, LTP was elicited by applying a theta-burst stimulation protocol and followed for an hour. The average of the peak-to-peak amplitudes of fEPSPs before the LTP induction was taken as 100%. The slices obtained from HCBS-injected animals showed robust potentiation after TBS (230 ± 24%; *n* = 15 channels from 5 slices). There were two more groups of icv injected animals treated with 24 h and 168 h Aβ1-42 aggregates. The 24 h Aβ1-42 assemblies caused a minor impairment in LTP (184 ± 7%; *n* = 26 channels from 6 slices), while the 168 h amyloid aggregates led to a major disruption of potentiation (145 ± 11%; *n* = 21 channels from 6 slices).

### 4.7. Statistical Analysis

Statistical analysis of MEA recordings: all data were expressed as the mean ± SEM. Statistical significance was determined by parametric analysis of one-way ANOVA followed by Fisher’s LSD post hoc test for LTP using the statistical software OriginPro 8 package (Origin Lab, Northampton, MA, USA). Differences with a *p*-value of less than 0.05 (*), 0.01 (**) and 0.001 (***) were considered significant. Statistical analysis of the Morris water maze experiment (Figure 9 and Figure 10.) was performed using SPSS software and Python’s Lifelines library (Davidson-Pilon, C., Lifelines, 2016), Github repository. The latency time to attain the platform (with 90 s limit) was measured in order to decide which of the four groups has the highest learning rate. The longer the latency time to the platform is, the less the rats’ capability to learn. “Survival curves” using the Cox Proportional Hazard model were fitted, where the days and the treatments were selected as covariates. The comparison of treatment groups was performed using log-rank tests.

One-way ANOVA followed by Fisher’s LSD post hoc test was used for histological analysis, for dendritic spine density measurements and for LTP analysis using SPSS statistical software. Differences with a *p*-value of less than 0.05 were considered significant unless indicated otherwise.

## Figures and Tables

**Table 1:** Variation of aggregation time and concentration for systematic studies of the effect on toxic Aβ1-42 aggregates used in biological experiments.

Groups of Aβ-Treated Animals	Aggregation Time (Hour)	Concentration (μM) of Aβ during Aggregation
A (*n* = 11), 24 h/25 μM	24	25
B (*n* = 11), 24 h/75 μM	24	75
C (*n* = 11), 24 h/200 μM	24	200
D (*n* = 12), 168 h/25 μM	168	25
E (*n* = 12), 168 h/75 μM	168	75
F (*n* = 12), 168 h/200 μM	168	200

The control groups (*n* = 11 and *n* = 12, respectively) got icv HCBS solution.

## Abbreviations

ACSF	Artificial cerebrospinal fluid
AD	Alzheimer’s disease
AFM	Atomic force microscopy
AMCA	7 Amino-4-methylcoumarin-3-acetic acid
ANOVA	Analysis of variance
ApoE	Apolipoprotein E
APP	Amyloid precursor protein
Aβ	Beta-amyloid
CA	Cornu Ammonis
EOAD	Early-onset AD
ER	Endoplasmic reticulum
fAβ	Fibrillar form of Aβ
fEPSP	Field excitatory postsynaptic potential
HC	Hippocampus
HCBS	Hydrocarbonate buffered saline
icv	Intracerebroventricular
LOAD	Late-onset AD
LTP	Long term potentiation
MEA	Multi-electrode array
MWM	Morris water maze
NFTs	Neurofibrillary tangles
oAβ	Oligomeric form of Aβ
PBS	Phosphate-buffered saline solution
PCA-AD	Posterial cortical atrophy variant of AD
PSEN1, 2	Presenilin-1 and 2
r-AD	Rapidy progressive form of AD
t-AD	Typical prolonged-duration form of AD
TBS	Theta-burst stimulation
